# Acute cell‑death and lysosomal stress responses to nicotine and cigarette smoke extract in human mesenchymal stromal cells

**DOI:** 10.1007/s00204-026-04440-w

**Published:** 2026-05-20

**Authors:** Anni Junttila, Janne Heikkinen, Elina Kylmäoja, Ayman Hawash, Pirjo Åström, Petri Lehenkari, Sanna Palosaari

**Affiliations:** 1 Translational Medicine Research Unit, University of Oulu, Oulu, Finland; 2 Research Unit of Biomedicine and Internal Medicine, University of Oulu, Oulu, Finland; 3 Biocenter Oulu, University of Oulu, Oulu, Finland; 4 Infotech Oulu, University of Oulu, Oulu, Finland; 5 Medical Research Center, University of Oulu and Oulu University Hospital, Oulu, Finland; 6 Division of Orthopedic Surgery, Oulu University Hospital, Oulu, Finland

**Keywords:** Mesenchymal stem cells, Cigarette smoke, Nicotine, Apoptosis, Necrosis

## Abstract

Mesenchymal stromal cells (MSCs) are essential for connective tissue repair, and impaired healing is well documented in tobacco users. MSCs are one plausible target for these adverse effects, but the underlying cellular mechanisms of high localized nicotine exposure remain poorly understood. This study investigated how short-term nicotine and cigarette smoke extract (CSE) exposure affect human MSC function, viability, and inflammatory signaling in vitro. MSCs isolated from bone marrow were exposed to CSE containing 4–40 µM nicotine or to 100 µM–10 mM pure nicotine. CSE produced markedly stronger cytotoxicity than nicotine, reducing proliferation and rapidly inducing necrotic cell death at 20–40 µM nicotine equivalents. Pure nicotine elicited a biphasic response: concentrations below 5 mM slightly increased proliferation, while 5 mM caused apoptotic cell death with prominent lysosomal vacuolization, and 10 mM shifted cell death toward necrosis. Sublethal exposures that generated pre-apoptotic cells were associated with significant IL8 induction and MMP2 activation, whereas IL6 remained largely unchanged. Nicotine induced lysosomal disruption suggests broader impacts on MSC homeostasis beyond viability, potentially influencing lineage commitment. These findings elucidate short-term effects of nicotine and CSE; high-dose nicotine and CSE are toxic to MSCs, while the lower doses perturb the inflammatory signaling and lysosomal function. Such alterations may compromise tissue regeneration, wound healing, and periodontal stability in users of potent localized nicotine delivery products.

**Supplementary Information:**

The online version contains supplementary material available at https://doi.org/10.1007/s00204-026-04440-w.

## Introduction

The use of tobacco free nicotine products, such as locally administrated pouches is increasing (Finnish Institute for Health and Welfare THL 2025). On mucosal layers, their use creates an environment where epithelial cells and the tissue regenerating mesenchymal stromal cells (MSCs) are locally exposed to increasing concentrations of nicotine. The constant use of nicotine pouches may cause localized gingival recession, leukoplakia and mucosal pathology (Alkhatib 2025). Gingival recession and root exposure leads to tissue and bone destruction. The concentrations of nicotine can be as high as 50 mg per pouch (Bundesinstitut für Risikobewertung 2022) and thus, the local nicotine concentration may rise considerably. However, the nicotine levels in tissues beneath the oral mucosa, such as the alveolar bone, and its cellular effects has remained unknown.

In smokers’ blood the levels of nicotine range from 0.4 to 1.3 µM but can be as high as 3–6 µM (Mano and Palani 2023).The concentrations in smokers’ saliva can range between 0.1 and 2 µM, peaking at 3 µM immediately after smoking (Feyerabend et al. 1982; Hukkanen et al. 2005). Even with these low nicotine concentrations long-term effects on mesenchymal cell function have been clearly indicated. Smoking increases the risk of osteoporosis and fractures, decreases bone mineral density, and impairs wound healing (Chang et al. 2020). In the oral region, smoking exacerbates periodontal diseases, increases alveolar bone loss, leading to tooth loss, and impairs dental implant treatments (Sharma et al. 2024).

The tissue responses to nicotine include several cell types. In general, nanomolar concentrations consistently increase cell growth, but the responses to micro to millimolar concentrations are more dependent on the cell type and other study dependent variables. On cell level nicotine effects on bone and connective tissue can be explained by mesenchymal cell responses. As we and others have shown previously (Kim et al. 2012; Shen et al. 2013; Heikkinen et al. 2024a) nicotine disturbs MSC growth and differentiation at sublethal concentrations. In micromolar range, nicotine effects vary depending on the MSC origin. In human alveolar bone marrow-derived MSCs, up to 100 µM nicotine concentrations had no effect on proliferation, while 1–2 mM nicotine increased proliferation but inhibited osteogenic differentiation (Kim et al. 2012). By comparison, on human bone marrow-derived MSCs below 600 nM nicotine exposure enhanced cell proliferation and osteogenic differentiation and over 1 µM nicotine significantly inhibited proliferation, increased apoptosis and inhibited osteogenic differentiation (Shen et al. 2013).

MSCs play a pivotal role in tissue regeneration, inflammation resolution, and maintaining structural integrity in various tissues, including the oral cavity and bone (Kangari et al. 2020; Nurul et al. 2021; Dipalma et al. 2025). Although the effects of cigarette smoke and nicotine on MSC biology have been previously investigated (Zeng et al. 2014; Heikkinen et al. 2024b), growing use of novel smokeless nicotine products such as nicotine pouches introduces new exposure dynamics that are not yet fully understood. The current study aims to shed light on the acute and subacute effects of high nicotine concentrations on MSCs, reflecting the local impact on the mesenchymal cells especially in the users of smokeless tobacco products.

## Materials and methods

### Human MSCs isolation and culture

MSCs isolated from bone marrow samples of six donors were used in this study (three males; ages 16, 41 and 44 years and three females; ages 17, 72 and 75 years). Bone marrow was collected during hip replacement surgery and MSCs were isolated and cultured as previously described (Leskelä et al. 2003). After removal of non‑adherent cells, adherent MSCs were expanded in α‑MEM (Corning, New York, USA) supplemented with 10% FBS (Biowest, Riverside, MO, USA), 20 mM HEPES (Corning, New York, USA), and 1% penicillin–streptomycin (Gibco, Waltham, MA, USA).

### Cigarette smoke extract and nicotine

CSE was prepared as previously described (Heikkinen et al. 2024b) by bubbling smoke from two North State cigarettes through 40 ml of culture medium. The extract was sterile‑filtered (0.2 µm), aliquoted, and stored at –80 °C. Nicotine content was quantified by mass spectrometry. For experiments, CSE and nicotine were diluted in basic medium, and MSCs were exposed for three days using concentration series in which the highest dose of both CSE and nicotine produced complete cell death within this period. The CSE exposure media was diluted to nicotine concentrations between 200 nM and 40 µM. For plain nicotine (EMD Millipore, Burlington, MA, USA) 200 nM to 10 mM exposure concentrations were used.

### Metabolic activity and cell viability

Metabolic activity was measured using the MTT assay (3‑[4,5‑Dimethylthiazol‑2‑yl] ‑2,5‑diphenyltetrazolium bromide; Methyl Thiazolyl Tetrazolium). MSCs from three donors were tested in three biological and five technical replicates (15 wells per group). After one and three days of nicotine or CSE exposure, cells were incubated for 4 h with 100 µl medium containing 0.5 mg/ml MTT. The solution was removed, and formazan was solubilized with 100 µl DMSO (Fisher Scientific, Waltham, MA, USA). Absorbance was measured at 550 nm with background subtraction at 650 nm using a Victor 2 plate reader (PerkinElmer Life Science/Wallac Oy, Turku, Finland). Values were normalized to 1‑day controls and expressed as fold change.

### Apoptosis and necrosis assay

The CSE and nicotine induced apoptosis and necrosis were quantitated by using Annexin V red and Cytotox green (IncuCyte, Essen BioScience, Sartorius) stains respectively. MSCs were seeded on 96-well plates and attached overnight. Annexin V red and Cytotox green stains were added on the cells with basic medium (controls), CSE or nicotine exposure medium (experimental groups). The cells were imaged using IncuCyte S3 Live-Cell imaging system (Essen BioScience, Sartorius) every two hours for 72 h and analyzed for phase confluence and green and red object count using Incucyte software version 2023A. All experiments were conducted as three biological and 3–6 technical replicates (162 wells were analyzed).

### PHrodo

For pHrodo assays, MSCs were seeded on 96-well plates at a density of 1.5 × 10^3^ cells per well and attached overnight. Cells were maintained in basic medium (controls) or exposed to 0.1–10 mM nicotine for 24 h. At 24 h culture medium was replaced with the pHrodo (Life Technologies, Canada, USA) solution with following protocol: 5 μL of pHrodo AM Ester was mixed with 50 μL of PowerLoad concentrate and 1.25 mL of basic medium. The cells were placed in the IncuCyte S3, incubated for 30 min and imaged once at 20 × using phase contrast and red fluorescence channels. The images were analyzed for red area confluence and red area integrated intensity using Incucyte software version 2024B. Experiments were performed as 3 biological and 5 technical replicates (15 wells per group, 90 wells were analyzed).

### LAMP1 staining

MSCs with and without exposure to 5 mM nicotine were fixed at a 24 h timepoint with 4% PFA-PBS and stored at 4 °C until further analysis. Cells were permeabilized with 0.1% Triton X-100-PBS for 10 min and blocked with 0.2% bovine serum albumin (Sigma-Aldrich) for 30 min at room temperature (RT). Cells were stained with 1:250 polyclonal rabbit LAMP1-antibody IgG (PA1—654, Invitrogen, Thermo Fisher Scientific, Waltham, MA, USA) for 1 h, at RT and labelled with anti-rabbit IgG Alexa 488 secondary antibody (Molecular Probes, Oregon, USA) diluted 1:100 in PBS for 1 h, at RT. The actin cytoskeleton was visualized with TRITC-conjugated phalloidin (Sigma-Aldrich, Merck KGaA, Darmstadt, Germany) 1:100 in PBS for 20 min at + 37 °C. Nuclei were stained with Hoechst 33258 (Sigma-Aldrich) 1:500 in PBS for 10 min at RT and mounted with ImmuMount (Thermo Fisher Scientific, Waltham, MA, USA). The staining was visualized with Leica Stellaris 8 DIVE confocal with a DMI8 microscope (Leica, Wetzlar, Germany) using LAS X 4.5.0 acquisition software (Leica, Wetzlar, Germany). The objective used was an HC PL APO CS2 20x/0.75 DRY. Samples were imaged with 405, 488 and 561 nm solid-state lasers with emission windows at 420–501, 501–562 and 566–706 nm. The pinhole was set to Airy 0.87 AU and scan speed to 600 Hz, line average was 4 and pixel size 0.57 µm.

### Transmission electron microscopy

For transmission electron microscopy 400 000 MSCs with or without 5 mM nicotine exposure for 24 h were fixated in 4% paraformaldehyde and 1% glutaraldehyde in 0.1 M phosphate buffer and pelleted by centrifugation. The cell pellets were post-fixed with osmium tetroxide, dehydrated using increasing concentration of acetone and embedded in plastic resin (Epon). Ultrathin Sects. (70–80 nm) were cut and post-stained with uranyl acetate and lead citrate before observation and imaging with Tecnai G2 Spirit 120 kV TEM with Veleta and Quemesa CCD cameras at Biocenter Oulu Electron Microscopy Core.

### Zymography

Gelatin zymography was performed to analyze the activity of matrix metalloproteinase (MMP)-2 and MMP-9. MSCs were seeded on 96-well plates at a density of 1000 cells per well. After overnight attachment, cells were exposed 1 µM to 10 mM nicotine and media were collected for zymography after 24 h from MSCs with and without nicotine exposure.

10 µl of each sample and positive controls (pro and active forms of MMP-2 and MMP-9) were mixed with 4 × sample loading buffer (250 mM Tris–HCl, 1.92 M glycine, 10 mM BPB) and loaded with a protein ladder PageRuler Plus (Thermo Scientific, Vilnius, Lithuania) on 10% sodium dodecyl sulfate (SDS)-polyacrylamide gels and 1% of fluorescence-labelled gelatin conjugated with 2-methoxy-2,4-diphenyl-3(2H)-furanone (MDF-gelatin). After electrophoresis (at 150 V for 1.5 h) the gels were washed once for 15 min and twice for 5 min in a buffer containing 2.5% Triton-X-100, 0.02% NaN_3_ in distilled water to remove SDS.

Following the wash steps, gels were incubated in a reaction buffer (50 mM TRIS, 5 mM CaCl2 2 × H2O, 1 µM ZnCl2) at 37 °C for periods ranging from 3 h to overnight to allow for gelatin degradation by MMPs. Gelatin degradation was visualized under long wave UV light using an AZUR 600 imaging system, with clear bands against a fluorescent background indicating proteinase activity. Images were analyzed, and the signal was quantified using Image Studio Lite software.

### IL-6 and IL-8 analysis

IL-6 and IL-8 were analysed from the culture medium after three days of exposure using IL-6 and IL-8 ELISA kits (Invitrogen Waltham, MA, USA) according to the manufacturer’s instructions. Experiments were performed as three biological and three technical replicates (9 wells per group, 117 wells analyzed for each cytokine). Results were normalized to control group by division and expressed as fold change.

### Statistical methods

SPSS statistics software, version 25 (IBM Corp., Armonk, NY, USA), was used for statistical analysis. The Kruskal–Wallis test was employed to investigate statistically significant differences between the test groups, while the Mann–Whitney U-test was used for pairwise comparisons. The direction and strength of dose responses were analyzed using Spearman’s correlation, with p-values for the correlation coefficient (R) calculated using the t-test. All tests were two-tailed, and *p*-values < 0.05 were considered statistically significant. No adjustments were made for multiple comparisons.

## Results

### Cell fate and inflammatory signalling in MSCs under nicotine and CSE-induced stress

Initially, the tolerance limits for both CSE and nicotine were established using MTT and apoptosis–necrosis assays. Within three days of exposure to CSE or nicotine, clear changes in MSC metabolic activity, confluency, cell death, morphology, and cytokine secretion were observed. In general, MSCs tolerated considerably higher concentrations of nicotine alone (up to 5 mM) than CSE (4 µM) before showing effects on mitochondrial activity or cell confluency.

**Fig. 1:**
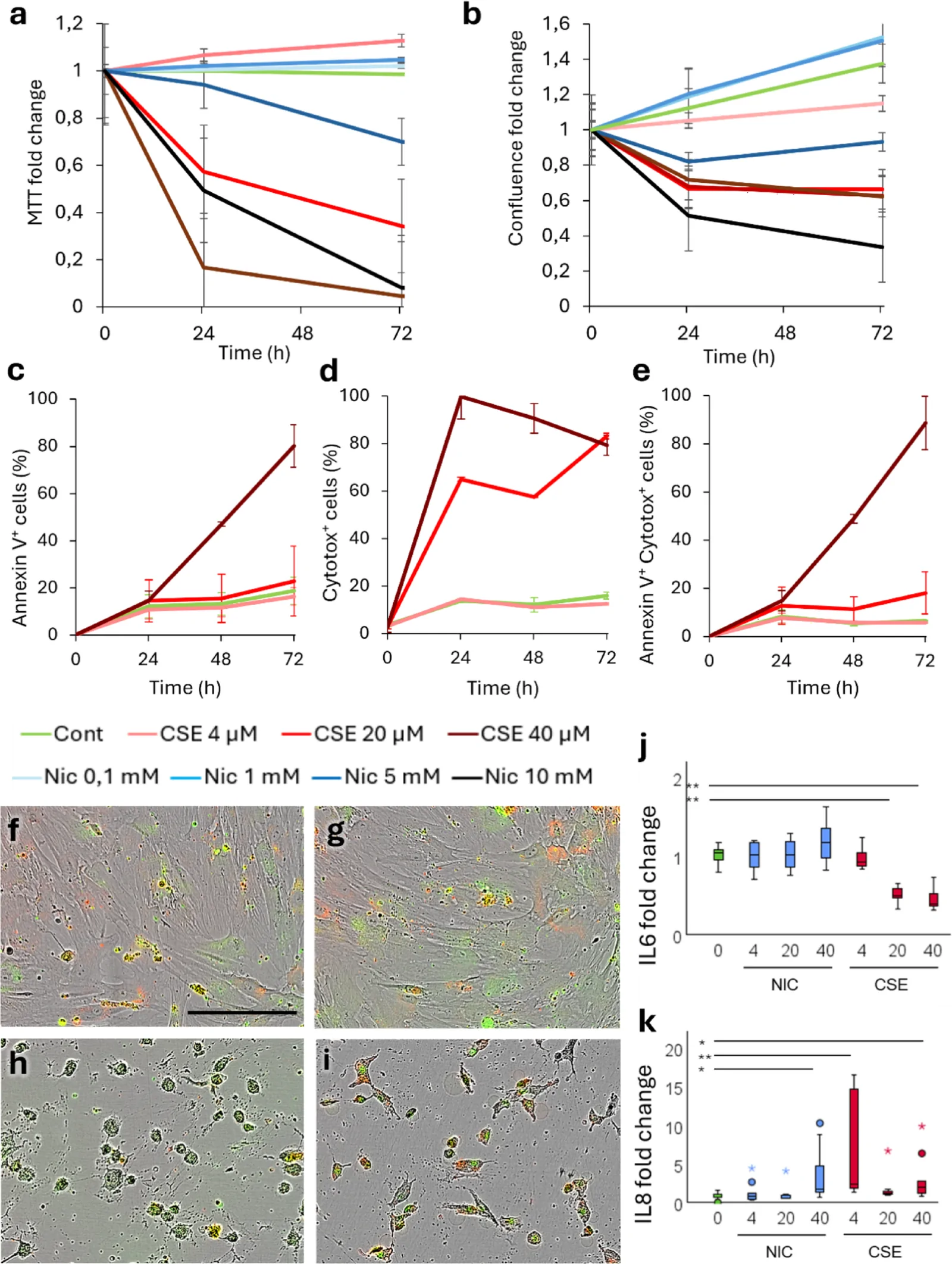
Nicotine and CSE show dose‑dependent effects on MSC metabolic activity, confluence, and cell death**. a** Metabolic activity decreased in a dose‑dependent manner for both nicotine and CSE over three days (MTT assay). **b** CSE and high nicotine concentrations reduced cell confluence, whereas low nicotine doses slightly increased it. CSE concentrations are shown as nicotine‑equivalent µM values. Data: mean ± SD from three experiments (n = 15/group). **c** Percentage of Annexin V–positive apoptotic cells, **d** Cytotox‑positive necrotic cells, and **e** Annexin V/Cytotox double‑positive cells (secondary necrosis) after three days under CSE exposures. Data: mean ± SD (n = 9–12/group). **f**–**i** Representative images of Annexin V (red) and Cytotox (green) staining for **f**) control, **g** CSE 4 µM, **h** 20 µM, and **i** 40 µM (scale bar = 200 µm, 10 ×). **j, k** Fold change in IL‑6 and IL‑8 secretion after three days of CSE or equivalent nicotine exposure. Statistical differences were assessed using Kruskal–Wallis with Mann–Whitney U tests (*p* < 0.05 considered significant). Boxplots show median, 25th–75th percentiles, whiskers (min–max), ° outliers, and * extreme outliers

MSCs showed clear concentration‑ and time‑dependent responses to both CSE and nicotine (Fig. 1a–b). CSE at 4 µM slightly increased metabolic activity (7% at day 1; 13% at day 3), but concentrations > 4 µM caused a rapid decline. Metabolic activity decreased by 43% (20 µM) and 83% (40 µM) after one day, and by 66% and 95% after three days. Correspondingly, CSE above 4 µM reduced confluency, and 40 µM led to near‑complete detachment by day 3. Pure nicotine produced a different pattern. Nicotine at 0.1 mM and 1 mM had no effect on metabolic activity and increased confluency by 54% and 52% after three days. Higher doses decreased mitochondrial activity by 6% (5 mM) and 50% (10 mM) at day 1, and by 30% and 92% at day 3. Confluency followed the same trend, with 5 mM and 10 mM reducing confluence by 6% and 67% after three days.

CSE induced a clear dose‑ and time‑dependent increase in both apoptotic and necrotic cell death (Fig. 1c–e). After three days, Annexin V–positive cells increased from 19% in controls to 23% at 20 µM and sharply to 80% at 40 µM, while remaining near control levels at 4 µM (16%) (Fig. 1c). Cytotox Green positivity rose from 16% in controls to 83% at 20 µM and 79% at 40 µM, with minimal change at 4 µM (12%) (Fig. 1d). Double‑positive analysis (Fig. 1e) indicated that 20–40 µM CSE caused predominantly necrotic death, whereas 4 µM resulted mainly in viable or apoptotic cells. CSE also increased IL‑8 secretion at 4 µM (Fig. 1k), while IL‑6 remained unchanged except for a decline at high CSE concentrations due to loss of viable cells (Fig. 1j).

**Fig. 2:**
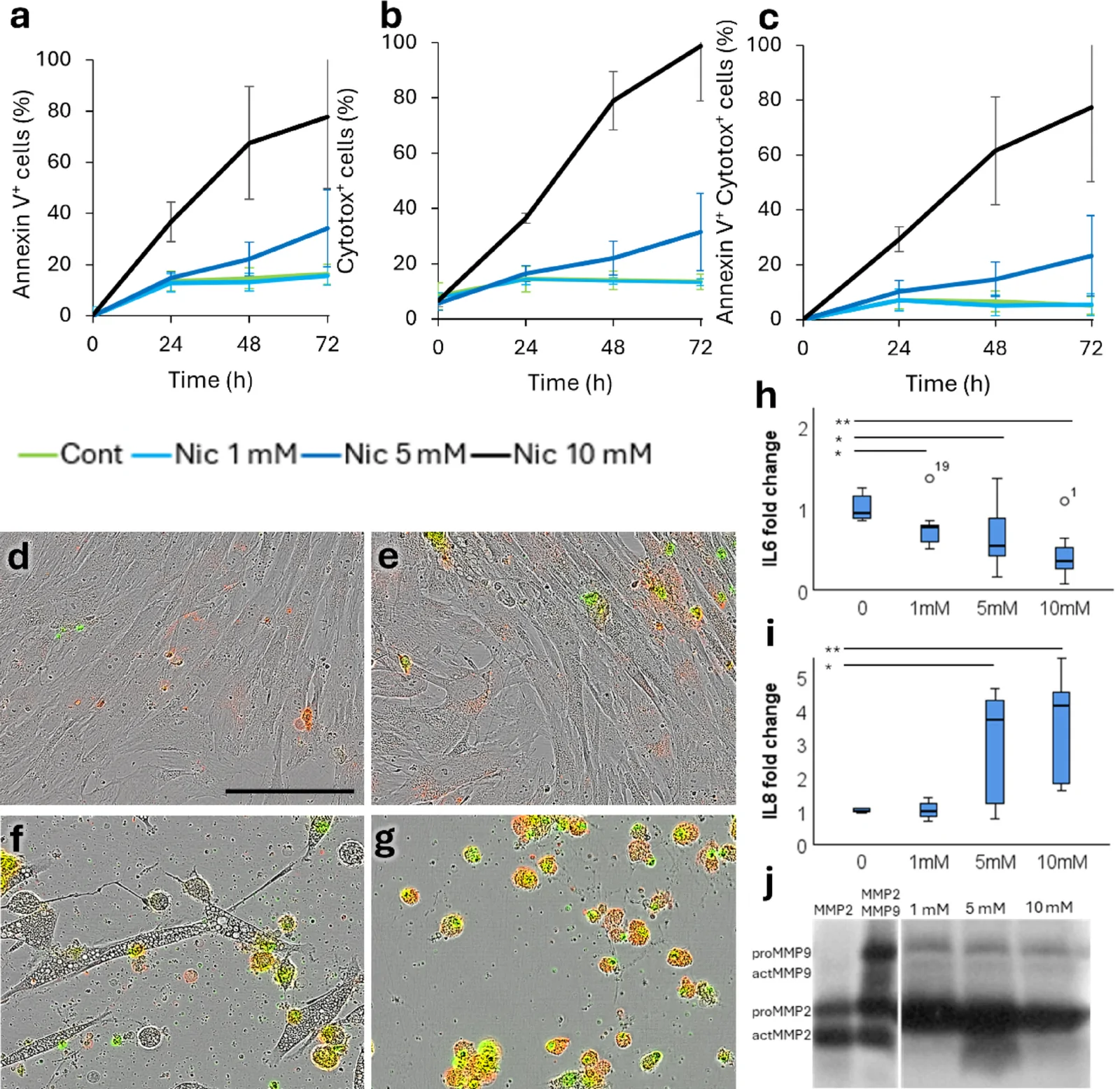
Nicotine induces dose dependent apoptosis in MSCs, and high doses elevate IL8 secretion**. a** Percentage of Annexin V–positive apoptotic cells, **b** Cytotox‑positive necrotic cells, and **c** Annexin V/Cytotox double‑positive cells (secondary necrosis) after three days of nicotine exposure. Data: mean ± SD from three independent experiments (n = 9–12/group). Representative images of Annexin V (red) and Cytotox (green) staining for **d** control, **e** 1 mM, **f** 5 mM, and **g** 10 mM nicotine (scale bar = 200 µm, 20 ×). **j** Gelatin zymography of conditioned media from nicotine exposed MSCs. **h, i** Fold change of IL6 and IL8 secretion after three days of nicotine or CSE exposure. Statistical analysis was performed with Kruskal–Wallis and Mann–Whitney U tests (*p* < 0.05 considered significant). Boxplots show median, 25th–75th percentiles, whiskers (min–max), ° outliers, and * extreme outliers

MSCs tolerated pure nicotine at far higher concentrations than CSE. Whereas CSE containing 40 µM nicotine killed all cells within 72 h, MSCs remained viable in 5 mM nicotine for the same duration. Nicotine induced cell death was mainly apoptotic up to 5 mM, with necrosis increasing only at 10 mM (17% at 48 h; 21% at 72 h, when all cells were dead). At 5–10 mM, Annexin V appeared before Cytotox Green, indicating secondary necrosis of apoptotic cells, while lower nicotine doses and controls showed distinct Annexin V and Cytotox signals. Low nicotine concentrations (0.1 mM, 1 mM) caused only modest apoptosis (≤ 17%) and necrosis (13%) after three days (Fig. 2a–c). Early stress signs, including cytoplasmic vacuolization, emerged within four hours of 5 mM exposure. By day 3, 5 mM nicotine produced 34% Annexin V–positive and 31% Cytotox positive cells. At 10 mM, Annexin V positivity rose to 78% and Cytotox to 99%, accompanied by cell rounding, vacuolization, and detachment (Fig. 2g). The high proportion of double positive cells (23% at 5 mM; 77% at 10 mM) supports apoptosis with secondary necrosis as the dominant death pathway, since only ~ 20% of cells were Cytotox only at the highest dose when all cells had died.

IL‑6 secretion decreased modestly at sublethal nicotine doses (0.1 mM, *p* = 0.004; 1 mM, *p* = 0.04) and was further suppressed at higher concentrations (5 mM, *p* = 0.04; 10 mM, *p* = 0.001; Fig. 2h). IL‑8 levels remained unchanged below 1 mM but increased significantly at 5 mM and 10 mM (5 mM, *p* = 0.04; 10 mM, *p* < 0.0001; Fig. 2i). Because nicotine induced marked but sublethal stress, we also examined MMP activity. MSCs expressed low pro‑MMP9 and abundant pro‑MMP2 under all conditions (Fig. 2j). Active MMP9 was not detected, whereas active MMP2 increased significantly under 5 mM nicotine exposure.

### Nicotine-induced changes in MSC morphology

**Fig. 3:**
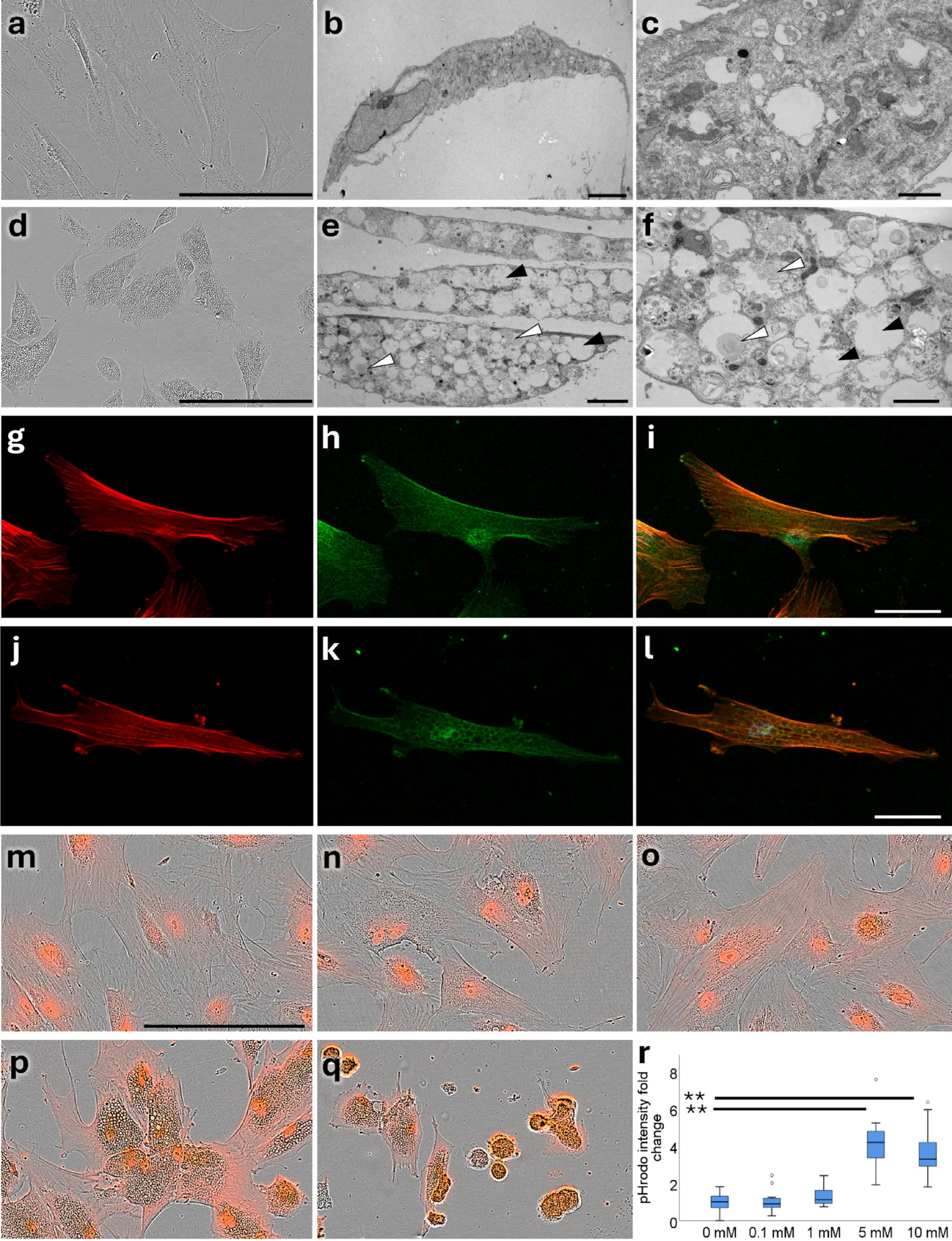
5 mM nicotine induces lysosomal vacuolization in MSCs**.** Representative images of control and nicotine‑exposed MSCs after 24 h. **a**–**c** Phase contrast and TEM images of control cells. **d**–**f** Corresponding images of MSCs exposed to 5 mM nicotine, showing extensive vacuolization with fluid filled lysosomes (black arrowheads) and myelin figures (white arrowheads). **g**–**i** Confocal images of control cells and **j**–**l** 5 mM nicotine‑exposed cells stained for actin (red) and LAMP‑1 (green). **m**–**q** IncuCyte images of pHrodo‑stained MSCs after 24 h exposure to nicotine: **m** control, **n** 0.1 mM, **o** 1 mM, **p** 5 mM, **q** 10 mM. **r** Fold change of pHrodo fluorescence intensity at 24 h. Data represents three independent experiments (n = 15/group). Statistical differences were assessed using Kruskal–Wallis with Mann–Whitney U for pairwise comparisons (*p* < 0.05 considered significant). Boxplots show median, 25th–75th percentiles, whiskers (min–max), ° outliers and * extreme outliers. Scalebars: live‑cell images A, D, M–Q = 200 µm; TEM B, E = 5 µm; C = 1 µm; F = 2 µm; confocal G–L = 50 µm

Since nicotine exposure led to distinct morphological alterations prior to detectable cell death, we further investigated these effects to better understand the nicotine induced cellular stress responses. Vacuolization appeared to be one of the earliest indicators of nicotine induced cell stress, preceding cell death. At control conditions and up to 1 mM nicotine concentrations, MSCs retained normal size, morphology, and a uniform distribution across the culture surface. These cells continued to proliferate and displayed no visual signs of stress during the 3-day exposure period, while exposure to 5 mM or 10 mM nicotine led to early and progressive morphological changes. Under 5 mM nicotine exposure cytoplasmic vacuolar structures started appearing immediately after exposure and became clearly visible within 4 h as shown in (Online Resource 1) as vesicles measuring 2–3 µm in diameter. By 24 h, the vesicles had expanded to their maximum observed size, with some reaching up to 11 µm in cross-section, and these features persisted throughout the 3-day observation period (Figs. 2f, 3d–f). Despite these changes, most cells remained viable throughout the experiment. Cells treated with 10 mM nicotine started forming vesicles similarly to 5 mM nicotine exposure and in addition to this the cells began to contract and round up, losing their typical spindle-shaped morphology. This was followed by widespread detachment from the plate surface, with most cells dying or detaching completely by day 3 (Fig. 2g).

We selected the 24 h timepoint on 5 mM nicotine exposure for transmission electron microscopy (TEM) analyses. In line with the live cell imaging (Fig. 3a and d), TEM images revealed prominent intracellular vacuoles in nicotine-exposed cells (Fig. 3e, f), which were absent in control cells (Fig. 3b, c). The vacuolar structures were lined with a single membrane and contained either membranous material (myelin figures) or an unstained liquid component, corresponding to lysosomal structures. Immunofluorescence staining of lysosome-associated membrane protein 1 (LAMP-1) confirmed these observations. In control cells, LAMP-1 staining appeared as small, dispersed vesicles (< 1 µm) throughout the cytoplasm (Fig. 3h) and 5 mM nicotine-treated MSCs showed LAMP-1 localization around enlarged vacuolar structures (Fig. 3k).

Finally, we confirmed the acidic nature of these vacuolar structures by live cell imaging with pHrodo staining after 24 h nicotine exposure. Again, at 0.1 mM and 1 mM nicotine exposure and under control conditions, cells maintained their morphology and pHrodo signal was faint and dispersed throughout the cytoplasm consistent with LAMP1 staining (Fig. 3m–o). However, cells exposed to 5 mM and 10 mM nicotine displayed a significant increase in pHrodo signal intensity and area (Fig. 3p, q). Compared to control cells, the red fluorescence integrated intensity was significantly elevated in MSCs exposed to 5 mM and 10 mM nicotine (Fig. 3r). The cell permeable pHrodo stained the vacuolar structures and confirmed these as enlarged lysosomes consistent with the findings in LAMP-1 and TEM analyses.

## Discussion

Here we demonstrated that CSE concentrations above 4 µM of nicotine were cytotoxic to undifferentiated MSCs while exposure up to 5 mM plain nicotine left the cells viable. Both sublethal CSE and nicotine exposure decreased IL6 secretion and increased IL8 secretion in MSCs. Interestingly, these sublethal doses of nicotine exposure induced significant changes in MSC morphology leading to lysosomal swelling. Exposure to low nicotine concentrations induced primarily apoptotic cell death, whereas 20 µM CSE and 10 mM nicotine exposures shifted the mechanism towards necrosis.

### Separate pathways for CSE and nicotine induced cell death

MSCs were far more sensitive to CSE than to pure nicotine. CSE containing 20 µM nicotine reduced viability, and 40 µM caused rapid necrosis, whereas the same nicotine concentrations in pure form had no cytotoxic effect. This is consistent with earlier reports of strong low-dose CSE toxicity (Aoshiba and Nagai 2003; Kagemichi et al. 2024), although direct comparison is complicated by the lack of quantified nicotine levels in those studies. In contrast, nicotine alone was tolerated up to millimolar levels and induced mainly apoptosis. Cells remained largely unaffected below 1 mM, while 5–10 mM reduced viability and shifted cell death from apoptosis (5 mM) to necrosis (10 mM). This pattern aligns with previous findings showing nicotine induced apoptosis only at millimolar concentrations (Zeng et al. 2014; Li et al. 2018) and high nicotine tolerance in MSCs and gingival fibroblasts (Kang et al. 2011). Similarly, earlier work has shown little cytotoxicity at concentrations up to 2 mM (Kim et al. 2012; Li et al. 2024), supporting our observation that the cytotoxic threshold of pure nicotine is substantially higher than that of CSE.

### Dose-dependent and biphasic effects of nicotine reflect tissue responses observed in nicotine pouch users

In nicotine pouch users, mucosal exposure to high local nicotine concentrations is sufficient to cause epithelial injury, and deeper tissue nicotine exposure is likely more subtle. A recent study (Miluna-Meldere et al. 2024), showed mucosal alterations—including inflammatory infiltration, edema, epithelial hyperplasia, and subepithelial fibrosis—at the site of pouch placement. Interestingly, deeper mucosal layers exposed to lower nicotine levels exhibited increased proliferation, resulting in acanthotic thickening. Consistent with our in vitro findings, nicotine exposure seems to induce both cell injury and proliferative activation in tissues. As we show here, plain nicotine induces a clear biphasic cellular response in MSCs. High concentrations (10 mM) led to widespread loss of viability within 72 h, whereas lower concentrations (0.1–1 mM) increased metabolic activity and confluence. At tissue level, the mode of cell death has consequences since apoptosis is resolved in anti-inflammatory manner, whereas necrosis releases DAMPs that activate stromal and epithelial cells and induce local inflammation.

Beyond DAMP mediated responses, nicotine and CSE exposed cells also secrete inflammatory cytokines that shape both autocrine and paracrine signaling. IL6 levels remained generally low across exposures, with only a slight, nonsignificant increase at very low nicotine concentrations (20–40 µM). Even modest IL6 elevation may, however, be biologically relevant: IL6 can promote survival under cellular stress by shifting energy metabolism toward a glycolytic, proproliferative phenotype (Kistner et al. 2022; Xu et al. 2024), potentially at the expense of MSC differentiation (Heikkinen et al. 2024b) and tissue homeostasis.

IL-8 secretion showed a clear, concentration-dependent increase following sublethal CSE and nicotine exposures. IL-8 not only attracts neutrophils and monocytes but also promotes proliferation of mesenchymal cells and keratinocytes (Rennekampff et al. 2000; Yang et al. 2018), thereby coordinating epithelial–mesenchymal–immune interactions during tissue injury. In keratinocytes, IL-8 enhances migration and context-dependent proliferation via CXCR2-mediated AKT/FOXO1 and PLC-γ signaling (Yu et al. 2024), while in mesenchymal cells it stimulates proliferation, migration, and VEGF-dependent angiogenic functions through PI3K/Akt and MAPK/ERK pathways (Hou et al. 2014; Yang et al. 2018). These mechanisms suggest that elevated IL-8 may simultaneously promote epithelial and stromal proliferation while sustaining inflammatory cell recruitment in nicotine-exposed tissues enriched in pre-apoptotic cells.

Although MSCs constitutively expressed MMP2, the active enzyme form was detected only in media from 5 mM nicotine exposed, preapoptotic cells with elevated IL8 levels. As IL8 can induce MMP2 expression through MAPK/ERK signaling (Pramanik and Mishra 2022), this suggests a link between nicotine induced cytokine release, matrix remodeling, and potential tissue injury or fibrosis. Nicotine induced MMP2 activation has been linked to aortic aneurysm development where MMP from vascular smooth muscle cells contributes to the degradation of extracellular matrix in the aortic wall (Wang et al. 2012; Rabkin 2016). Interestingly, the MSC derived MMP2 activation could contribute to the tissue permeability and degradation of mucosal and submucosal structures in nicotine pouch users.

### The role of lysosomal function in mesenchymal cell faith

Nicotine-induced apoptotic cell death in MSCs was preceded by pronounced cytoplasmic vacuolization under 5 mM exposure. Within 24 h, MSCs accumulated large vacuolar structures identified as lysosomes by LAMP1 staining, transmission electron microscopy, and pHrodo live-cell imaging. Lipophilic weak bases such as nicotine diffuse freely into the cytoplasm and accumulate in acidic organelles like lysosomes (Aki et al. 2012). Protonation within lysosomes traps nicotine and promotes osmotic swelling (Li et al. 2024), explaining the observed vacuolization. Nicotine has previously been linked to lysosomal swelling, autophagy induction, and cytoplasmic vacuolation in several cell types (Kang et al. 2011; Zeng et al. 2014; Du et al. 2021; Rinaldi et al. 2023). Alongside vacuolization, nicotine can impair lysosomal degradative capacity and increase p62/SQSTM1 and LC3-II, indicating disrupted autophagic flux (Uvsløkk et al. 2025).

Lysosomes are central regulators of cellular homeostasis. Beyond degradation, they coordinate energy metabolism, respond to stress, and modulate cell fate by maintaining membrane integrity and acidic pH (Scott et al. 2025). In aging MSCs, gradual lysosomal alkalinization impairs autophagy, leading to the buildup of damaged macromolecules and oxidative stress—processes that destabilize lysosomal membranes and promote senescence (Zhang et al. 2022). Nicotine appears to trigger a similar response as increased lysosomal pH compromises membrane stability and releases cathepsins such as cathepsin B into the cytosol (Wu et al. 2025). Notably, nicotine induced lysosomal vacuolation is reversible after 24 h exposure (Li et al. 2024), highlighting lysosomal plasticity at the boundary between survival and cell death (reviewed in Jung et al. 2020).

Lysosomal function also shapes MSC lineage decisions. Lysosomes regulate organelle turnover and proteomic remodelling through nutrient-sensing pathways such as mTORC1 and AMPK, which link metabolism to cell cycle control, self-renewal, and differentiation (Julian and Stanford 2020). Chaperone-mediated autophagy–dependent lysosomes have been shown to drive osteogenic differentiation (Gong et al. 2021). Vangl2 signaling promotes osteoblast commitment by mediating autophagic degradation of inhibitory regulators including ZNF423 and TLE3 via the Vangl2–LAMP-2A axis (Wei et al. 2024). Inhibition of autophagy shifts MSC commitment toward adipogenesis, whereas its activation enhances osteoblast differentiation. These mechanisms provide a compelling explanation for our previous findings on long-term nicotine effects on MSC differentiation (Heikkinen et al. 2024b). Micromolar nicotine does not induce visible lysosomal swelling, but even modest, chronic impairment of lysosomal function could contribute to the increased lipid accumulation in adipogenic conditions (Heikkinen et al. 2024a) and reduced type I collagen production in osteogenic conditions (Heikkinen et al. 2024b) observed after three weeks of exposure to 40 µM nicotine.

As study limitations, it should be noted that this study was conducted using in vitro assays, which cannot fully replicate the complex microenvironmental, metabolic, and immunological interactions that occur in vivo. As with all primary cell–based studies, donor variability introduces biological heterogeneity that may influence the results. In addition, the relatively small sample sizes reduce the statistical power and generalizability of the conclusions. Future studies should address these limitations by incorporating in vivo models, and larger cohorts to more accurately characterize the effects of nicotine and CSE on mesenchymal cell function.

## Conclusion

MSCs are sensitive for both nicotine and CSE, albeit the cellular responses may vary. While MSCs tolerated high nicotine concentrations and underwent predominantly apoptotic death, CSE lead to MSC necrosis. Both nicotine and CSE elevated IL-8 production in pre‑apoptotic cells, offering a mechanistic link to proliferative tissue changes observed in nicotine pouch users, including epithelial thickening and subepithelial fibrosis. In addition, nicotine-induced lysosomal dysfunction indicates broader impacts on MSC behavior, potentially affecting lineage commitment alongside proliferation and cell survival.

Together, these findings show that localized nicotine exposure can disrupt tissue homeostasis and MSC driven regeneration through combined effects on inflammatory signaling, lysosomal regulation, and cell fate decisions. Such alterations may impair mesenchymal cell–dependent processes—including wound repair, periodontal stability, and bone remodeling—underscoring the need for careful evaluation of high-dose nicotine delivery products.

## Supplementary Information

Below is the link to the electronic supplementary material.

Supplementary Material 1.

## Data Availability

The datasets generated and analysed during the current study are available from the corresponding author on reasonable request.
