# Supplementary material for: Acute cell‑death and lysosomal stress responses to nicotine and cigarette smoke extract in human mesenchymal stromal cells
Source: Arch Toxicol. 2026 May 20;100(10):4633–44. doi: 10.1007/s00204-026-04440-w (PMC13593743; doi:10.1007/s00204-026-04440-w)
Supplement: Supplementary file 1 [file 204_2026_4440_MOESM1_ESM.pdf]

Archives of Toxicology

Acute cell-death and lysosomal stress responses to nicotine and cigarette smoke extract in human mesenchymal stromal cells

Anni Junttila[1*], Janne Heikkinen[1*], Elina Kylmäoja[1], Ayman Hawash[2,3], Pirjo Åström[2,3,4] Petri Lehenkari[1,5,6#], Sanna Palosaari[1,5#]

[1] Medical Faculty, Translational Medicine Research Unit, University of Oulu, Oulu, Finland

[2] Research Unit of Biomedicine, University of Oulu, Oulu, Finland

[3] Biocenter Oulu, University of Oulu, Finland

[4] Infotech Oulu, University of Oulu, Finland

[5] Medical Research Center, University of Oulu and Oulu University Hospital, Oulu, Finland

[6] Division of Orthopedic Surgery, Oulu University Hospital, Oulu, Finland

[*, #] equal contribution

Anni Junttila, anni.junttila@oulu.fi ORCID: 0009-0001-6151-9338

Janne Heikkinen, janne.heikkinen@oulu.fi ORCID: 0000-0002-7787-908X

Elina Kylmäoja, elina.kylmaoja@oulu.fi ORCID: 0000-0003-0821-0718

Ayman Hawash, Ayman.Hawash@oulu.fi ORCID: 0009-0002-2196-7563

Pirjo Åström, pirjo.astrom@oulu.fi ORCID: 0000-0001-7150-2508

Petri Lehenkari, petri.lehenkari@oulu.fi ORCID: 0000-0002-0055-5183

Sanna Palosaari, sanna.palosaari@oulu.fi ORCID: 0000-0003-1526-5784

Corresponding author:

Anni Junttila, anni.junttila@oulu.fi ORCID: 0009-0001-6151-933, phone +358442829642

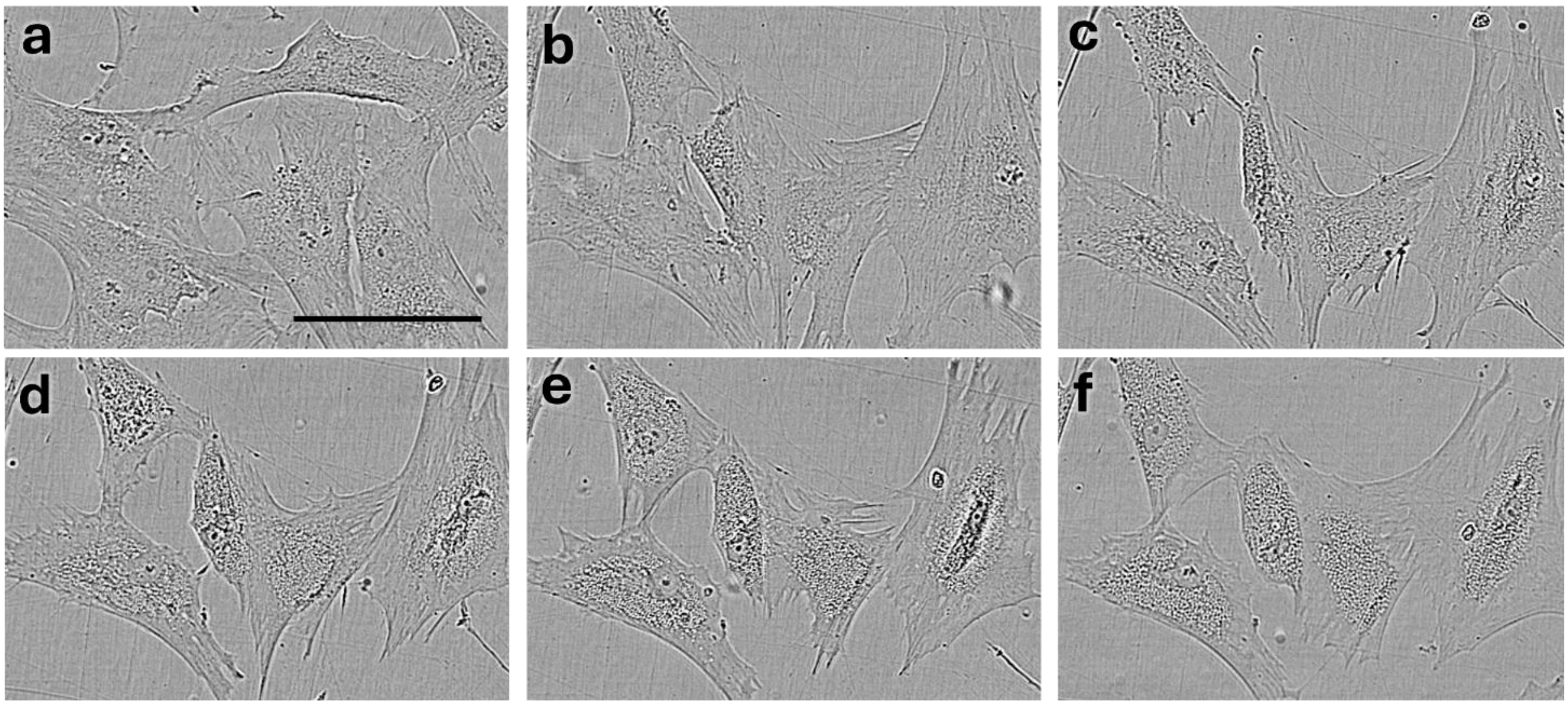

Online Resource 1 Progressive lysosomal vacuolization of MSCs. Pictures represent the vacualization of cells in nicotine concentration 5mM with varying time points. A is from control untreated cells and pictures B-F are exposed to nicotine. A and B are 0h, C is 30min, D is 60min, E is 2h and F is 4h. The scale bar is 200μm. Data is from a single MSC cell line.
